# Supplementary material for: Discovery of ultrafast spontaneous spin switching in an antiferromagnet by femtosecond noise correlation spectroscopy
Source: Nat Commun. 2023 Nov 29;14:7651. doi: 10.1038/s41467-023-43318-8 (PMC10687256; doi:10.1038/s41467-023-43318-8)
Supplement: Supplementary file 1 — Supplementary Information [file 41467_2023_43318_MOESM1_ESM.pdf]

Supplementary Information: Discovery of ultrafast spontaneous spin switching in an antiferromagnet by femtosecond noise correlation spectroscopy

M. A. Weiss[1], A. Herbst[1], J. Schlegel[1], T. Dannegger[1], M. Evers[1], A. Donges[1], M. Nakajima[2], A. Leitenstorfer[1], S. T. B. Goennenwein[1], U. Nowak[1] & *T. Kurihara[1,3]

[1]Department of Physics, University of Konstanz, D-78457 Konstanz, Germany.
[2]Institute of Laser Engineering, Osaka University, Japan.
[3]The Institute for Solid State Physics, The University of Tokyo, Japan.

SI1. Experiment: LF feature spectral amplitude versus temperature

The spectral amplitude of the LF peak as a function of temperature is depicted in Suppl. Fig.1. As in the temperature evolution of the time-zero peak (Fig. 3b), a strong noise enhancement is observed at $T \approx 312$ K $> T_{\mathrm{L}}$. In this temperature range, the spectral amplitude is an order of magnitude larger than the F-mode noise (see Suppl. Fig. 2). This finding suggests that random switching between two quasi-equilibrium states (see main text) is the major contributor to the observed total noise amplitude (Fig. 3b) for temperatures $T \gtrapprox T_{\mathrm{L}}$.

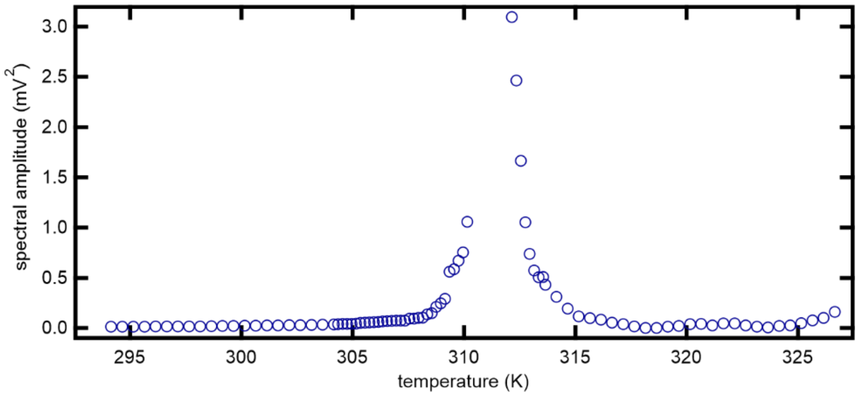

Suppl. Figure 1 | Spectral amplitude of measured LF peak in $Sm_{0.7}Er_{0.3}FeO_3$ as a function of temperature.

SI2. Theory: Simulated equilibrium properties of the orthoferrite

Supplementary Figure 2 shows the equilibrium properties of the simulated orthoferrite as a function of temperature. The equilibrium Néel vector n_β/n_S and magnetisation vector components m_β/m_S ($\beta = a, b, c$), normalised to the saturation values n_S and m_S, are obtained by taking the average of multiple simulation runs. The simulated system exhibits a transition to the paramagnetic state at Néel temperature at $T_{\mathrm{N,sim}} \approx 630$ K and furthermore shows a temperature-induced 2nd order reorientation transition in a finite temperature window, where the Néel and magnetisation vector rotate at $T_{\mathrm{L,sim}} \approx 301.5$ K from the *c*-axis and *a*-axis by 90° to the *a*- and *c*-axis at $T_{\mathrm{U,sim}} \approx 322$ K, respectively.

*Corresponding author: Takayuki Kurihara (takayuki.kurihara@issp.u-tokyo.ac.jp)

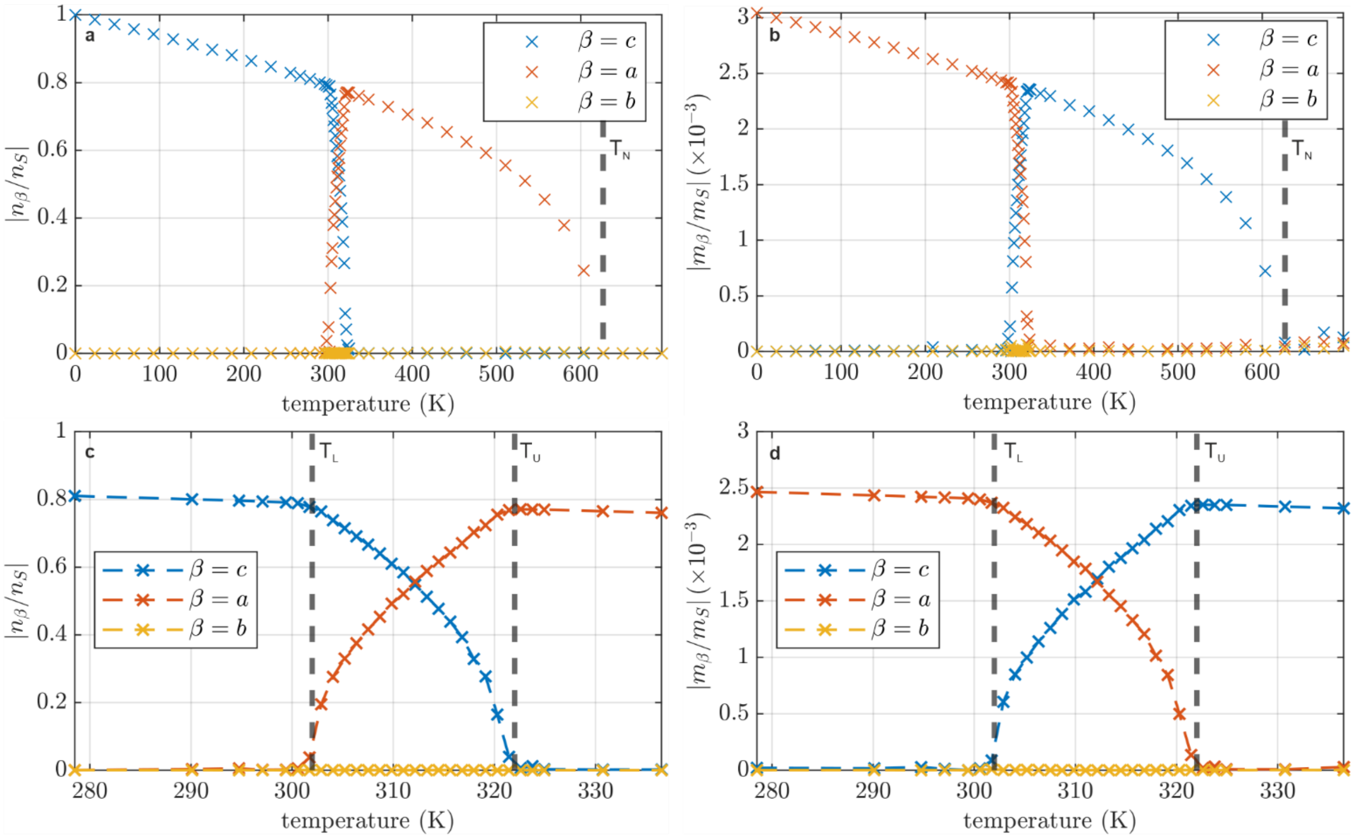

Suppl. Figure 2 | Equilibrium properties of the simulated orthoferrite. a,b, Néel vector n_β/n_S (**a**) and magnetisation vector components m_β/m_S (**b**) in a temperature range where the systems exhibits magnetic ordering. The dashed line indicates the tempearute $T_N \approx 630$ K at which the magnetic ordering is lost and the system transitions into a paramagnetic state. **c,d**, Néel vector n_β/n_S (**c**) and magnetisation vector components m_β/m_S (**d**) for multiple temperatures across the spin reorientation transition, where the Néel and magnetisation vector rotate at $T_{L,sim} \approx 301.5$ K from the *c*-axis and *a*-axis by 90° to the *a*- and *c*-axis at $T_{U,sim} \approx 322$ K, respectively.

SI3. Theory: Resonance modes in $Sm_{0.7}Er_{0.3}FeO_3$

The resonance modes of $Sm_{0.7}Er_{0.3}FeO_3$ at temperatures below the SRT are shown in Suppl. Fig 3. They were determined from the eigenfunctions of the linearised LLG equation. Below the SRT (low temperature regime, LT), the Néel vector is oriented along the *c*-axis, while is parallel to the *a*-axis above the SRT (high temperature regime, HT). For both temperature regimes, we see two sub-THz modes emerge with pairwise collinear sublattice magnetisations, as well as two multi-THz exchange modes with non-collinear sublattice magnetisation vectors. In terms of the net magnetisation vector, the modes can be grouped into qF modes, characterised by an elliptical trajectory of the magnetisation vector, and qAF modes, where the norm of the magnetisation oscillates along a fixed direction.

The supplementary videos 1-8 visualise the dynamics of the resonance modes in $Sm_{0.7}Er_{0.3}FeO_3$. As in Suppl. Fig 3, the arrows symbolise the sublattice magnetisation vectors, with the red arrow indicating the magnified net magnetisation vector and the purple arrow indicating the Néel vector. The modes are numbered 1 to 4 in order of their frequency (lowest to highest).

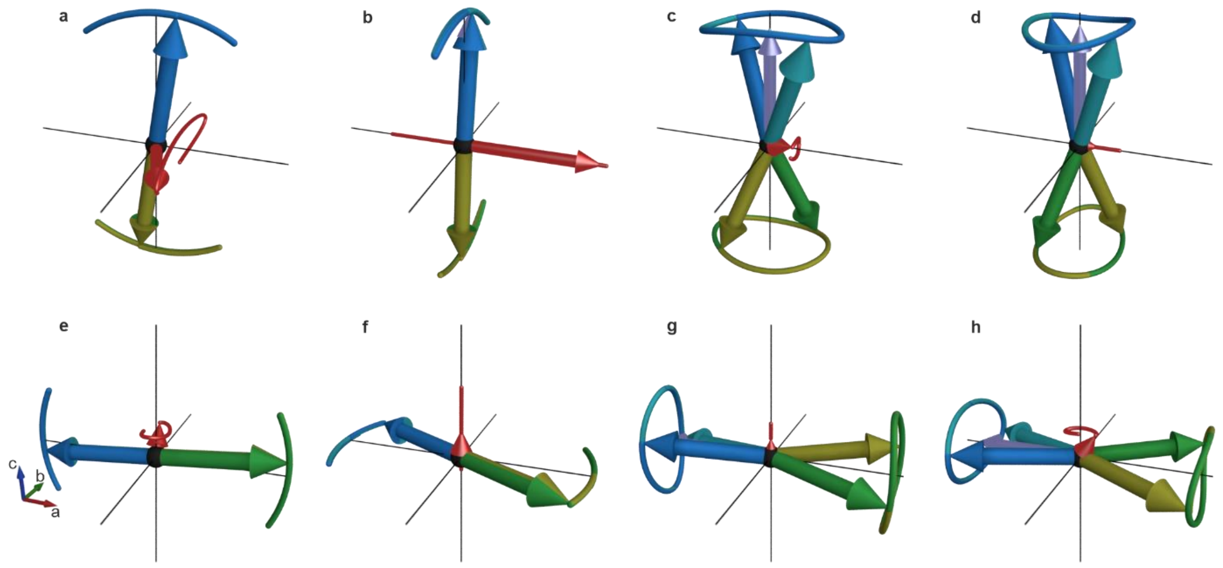

Suppl. Figure 3 | Resonance modes of $Sm_{0.7}Er_{0.3}FeO_3$ across the spin reorientation transition. a-d, Resonance modes in the low temperature regime (below the lower threshold temperature T_{L}). **e-f**, Resonance modes in the high temperature regime (above the upper threshold temperature T_{U}). The blue, yellow, mint, and green arrows symbolise the sublattice magnetisation vectors, while the purple arrow represents the Néel vector. The red arrow indicates the net magnetisation vector, for visibility magnified by a factor of 75 relative to the sublattice magnetisation vectors. The lines of the same colours represent the respective trajectories of the magnetisation vectors in time.

SI4. Theory: Extended data

Suppl. Fig. 4a-c shows the spectra of simulated spin noise projected along the crystalline axes for multiple temperatures across the SRT. In both, *a*- and *c*-axis projections up to three noise peaks can be distinguished, namely LF peak (<100 GHz), qF mode peak (100-200 GHz) and qAF mode peak (~600 GHz), whereas only the qF mode is observed in the *b*-axis projection. The spectral amplitude of the LF peak as a function of temperature is plotted in Suppl. Fig. 4d. Along the *a*-axis, the LF peak is observed for $T \gtrapprox T_{\mathrm{L,sim}} \approx$ 301.5 K and along the *c*-axis close to the upper threshold temperature $T_{\mathrm{U,sim}} \approx$ 320 K. The temperature ranges in which the LF peak is recorded precisely coincides with the observation of RTN in the magnetisation time traces (Suppl. Fig.5). This further supports our claim that random switching between two quasi-equilibrium states, resulting in picosecond RTN is the physical mechanism behind the emergence of the LF peak in the experiment. Furthermore, no LF peak and no RTN is observed in the *b*-axis projection of simulated spin noise. This is expected because the *b*-axis is the hard axis in $Sm_{0.7}Er_{0.3}FeO_3$. Consequently no quasi-equilibrium states arise in this direction at any temperature. Suppl. Fig. 4e shows the temperature evolution of the qF mode magnon's spectral amplitude for different crystalline axes. Most notably, a strong enhancement along the *c*-axis is observed close to $T_{\mathrm{L,sim}}$ in accord with the Fluctuation-Dissipation Theorem. For $T_{\mathrm{L,sim}} < T < T_{\mathrm{U,sim}}$, the SRT takes place and consequently the *c*-axis projection of the qF mode noise reduces as $\propto \cos(\theta)$. At $T_{\mathrm{U,sim}}$, the equilibrium magnetisation $\mathbf{M}$ is aligned, with the *c*-axis and no more transversal qF mode noise can be observed in the *c*-axis direction. At the same time, the qF mode noise in the *a*-axis direction is maximal at $T_{\mathrm{U,sim}}$. For all temperatures, the *b*-axis component of the magnetisation $m_b = 0$. Consequently, a transversal qF mode noise component is always observed in this direction, although at smaller amplitude compared to *a*- and *c*-axis, because the *b*-axis is the hard axis in our system. The latter is furthermore expressed as the ellipticity of noise cone in the trajectory plots (Fig. 5b-e).The longitudinal qAF mode noise can only be observed along a specific axis, if the equilibrium magnetisation component along the said axis is non-zero. As a consequence, no qAF mode noise is observed along the *b*-axis in Suppl. Fig. 4f. Furthermore, the qAF mode spectral amplitude in *a*-direction is maximal for $T < T_{\mathrm{L,sim}}$ ($\mathbf{M}$ // *a*), then reduces in the SRT region ($T_{\mathrm{L,sim}} < T < T_{\mathrm{U,sim}}$) until $\mathbf{M}$ // *c* for $T \geq T_{\mathrm{U,sim}}$. In contrast, the AF noise along the *c*-axis is first zero, then increases during the SRT and is maximal for $T \geq T_{\mathrm{U,sim}}$. We plot the centre frequency of the qF mode and qAF mode as a function of temperature in Suppl. Fig. 4g. In accord with previous observations[1], the qF mode experiences strong softening close to the SRT region, whereas only slight frequency changes of the qAF mode are observed. The above result evidences that our stochastic spin model can correctly reproduce our experimental observations and is consistent with physical expectations.

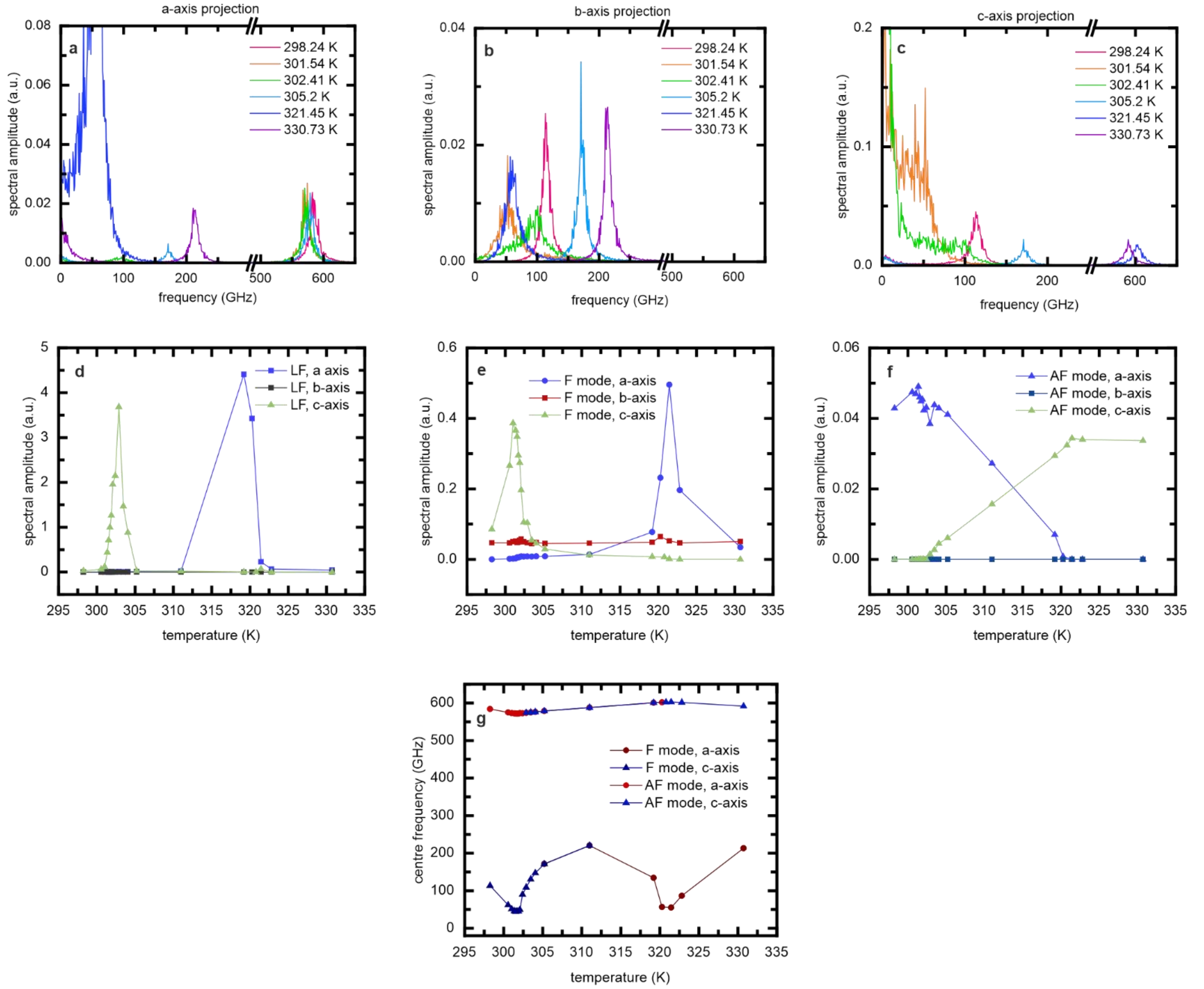

Suppl. Figure 4 | Spectra and evaluation of simulated spin noise in Sm$_{0.7}$Er$_{0.3}$FeO$_3$. for different projections. a-c, Raw spectra of simulated spin noise for different temperatures near spin reorientation. The peak at ~600 GHz is attributed to the quasiantiferromagnetic magnon mode (qAF mode), the middle peak, which shows a strong temperature dependence is attributed to the quasiferromagnetic mode (qF mode), and the low-frequency feature (LF) to random switching between two energetically degenerate quasi-equilibrium states. **d-f**, temperature evolution of the LF peak, qF mode, and qAF mode spectral amplitude for different axes. **g**, central frequency of qF mode and qAF mode as a function of temperature.

Supplementary Figure 5 shows different projections of the simulated magnetisation time traces. At temperatures $T \gtrsim T_{\mathrm{L,sim}}$ and $T \lesssim T_{\mathrm{U,sim}}$, switching events are recorded in the *c*- (Suppl. Fig 5c) and *a*-direction (Suppl. Fig 5a), respectively, due to anisotropy softening. For all temperatures, the fluctuations are centred around 0 in the *b*-axis projection. The attached supplementary video 9 shows all the simulated trajectories of the normalised magnetisation components m_β/m_S $(\beta = a, b, c)$. Per temperature, a total number of 20 time traces were simulated, each with a total length of 1300 ps.

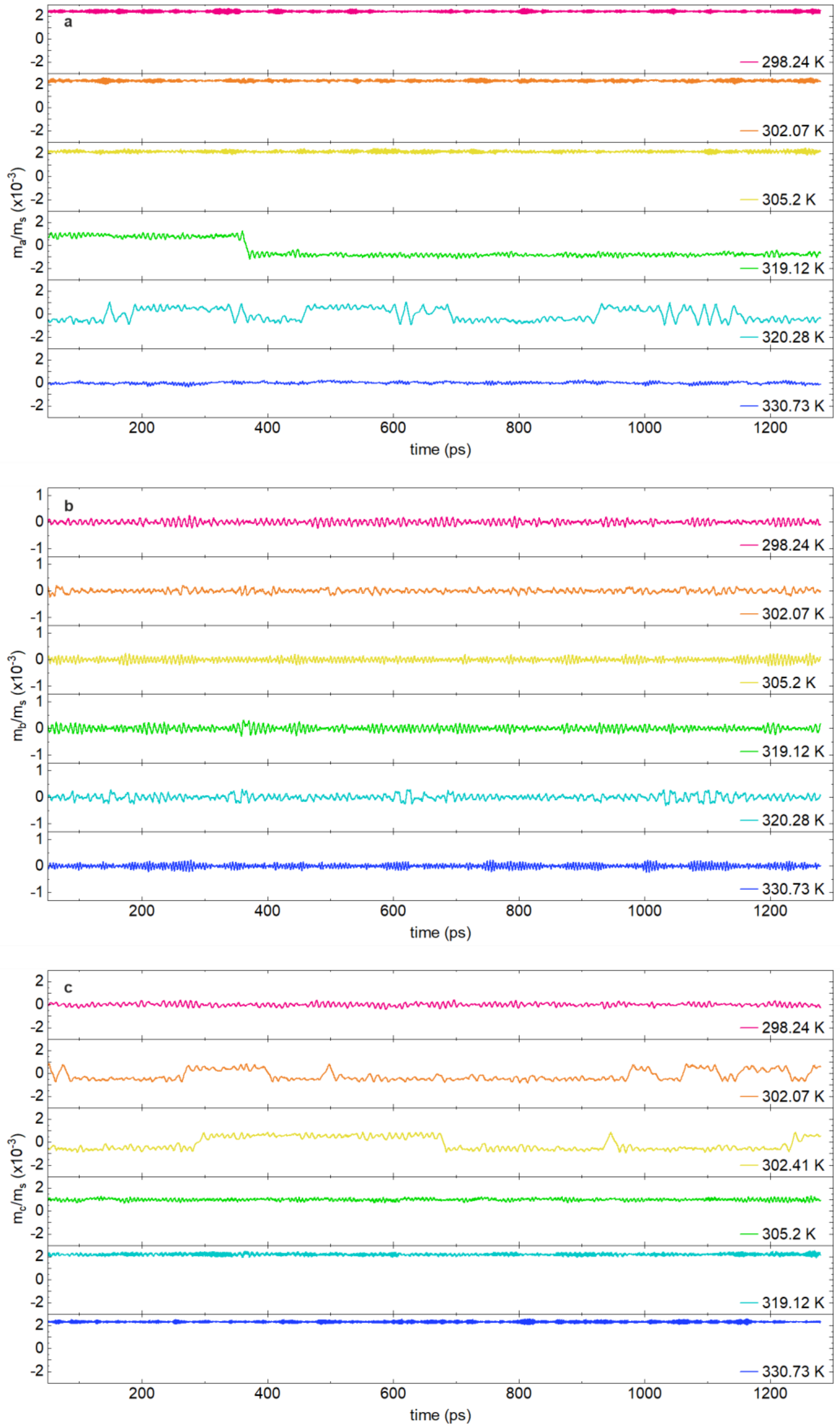

Suppl. Figure 5 | Simulated time traces of different components of the normalised magnetisation near spin reorientation in $Sm_{0.7}Er_{0.3}FeO_3$. a-c, normalised *a*-axis (**a**), *b*-axis (**b**) and *c*-axis (**c**) component of the magnetisation. For every temperature, only the first time trace from a total number of 20 simulated traces is shown. The first 50 ps are not shown, because here the system still equilibrates from the initial conditions.

SI5. Mean dwell time τ estimation of the random telegraph noise

Because the autocorrelation of a pure RTN signal is expected to decay as $\sim \left(\frac{\Delta m_c}{2}\right)^2 e^{-2\frac{\Delta t}{\tau}}$ (Ref. [2]), we estimate the mean dwell time τ RTN τ_{sim} by time-domain analysis of the raw magnetisation traces showing RTN and by exponential fitting of the correlation waveforms.

Time-domain analysis is conducted following the algorithm proposed by Yuzhelevski et al. (Ref. [3]). It utilises the difference in the statistical properties of the RTN (dichotomous Markov process) and normal distributed background fluctuations to assign all recorded data points to one of the two discrete RTN levels. Thus the pure RTN is distilled from other noise sources allowing independent analysis of the former. To obtain more accurate results, we artificially extend our time window by appending the data points of each simulation run belonging to the same temperature. The first 50 ps of each simulation run is neglected, because here the system equilibrates from the initial conditions of the simulation. The appended time traces serve as the input for our evaluation. Note, that this procedure may create artificial switching events. These, however, only have noticeable impact on time traces where the total number of switches is in the order of one event per simulation run. We then plot the histograms of the appended noisy RTN signals and employ double Gaussian fitting (Suppl. Fig 6) to obtain the statistics of the background noise. The fitting parameters are used as an input for the destillation algorithm which yields the pure RTN as shown in Suppl. Fig 6. Eventually, the mean dwell time τ_{sim} is obtained using the total number of switches and duration of the pure RTN signal[3]. The temperature evolution of the number of switches and the mean dwell time τ_{sim} obtained from time-domain analysis is plotted in Suppl. Fig 7b. We neglect the magnetisation time traces where no two levels are clearly distinguishible, i.e. where the Gaussians in the histogram show strong overlap, and thus the alrgorithm does not converge. We

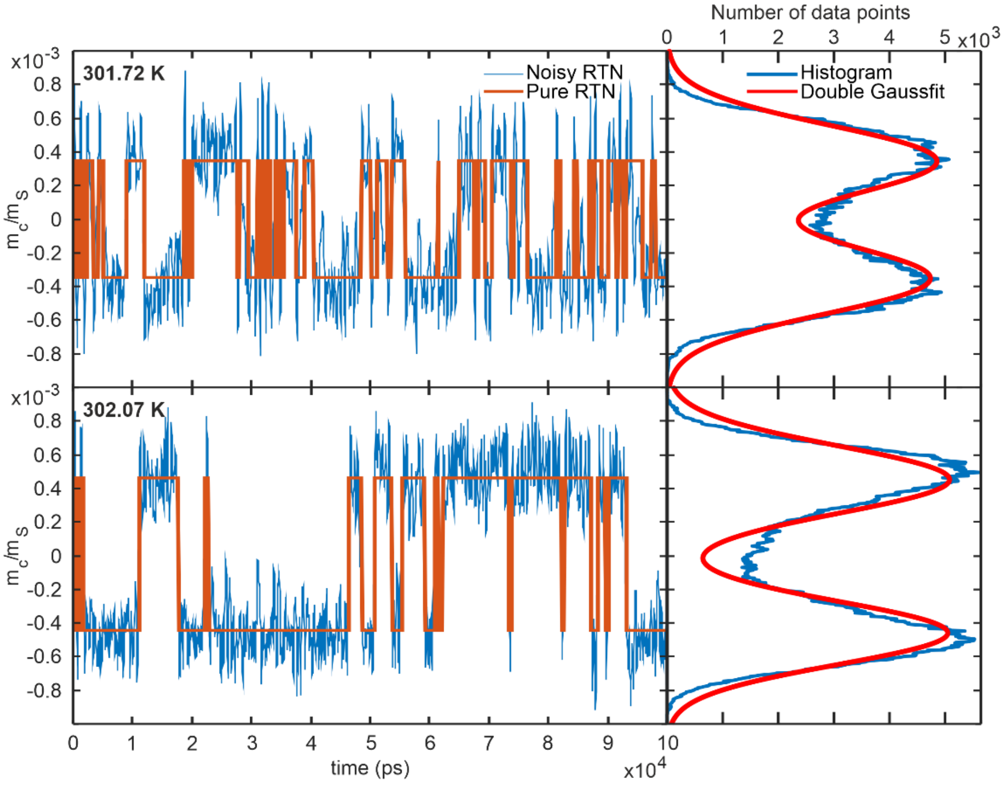

Suppl. Figure 6 | Visualisation of the random telegraph noise destillation algorithm. The histograms (right, blue line) of the simulated magnetisation time traces at temperatures 301.72 K and 302.07 K (left, blue line) are fitted with double Gaussian fits (right, red line) to extract the statistical properties of the background noise in the magnetisation time traces. Employing the algorithm as proposed in Ref. [3] then yields a pure two-level random telegraph noise time trace without background noise (left, orange line).

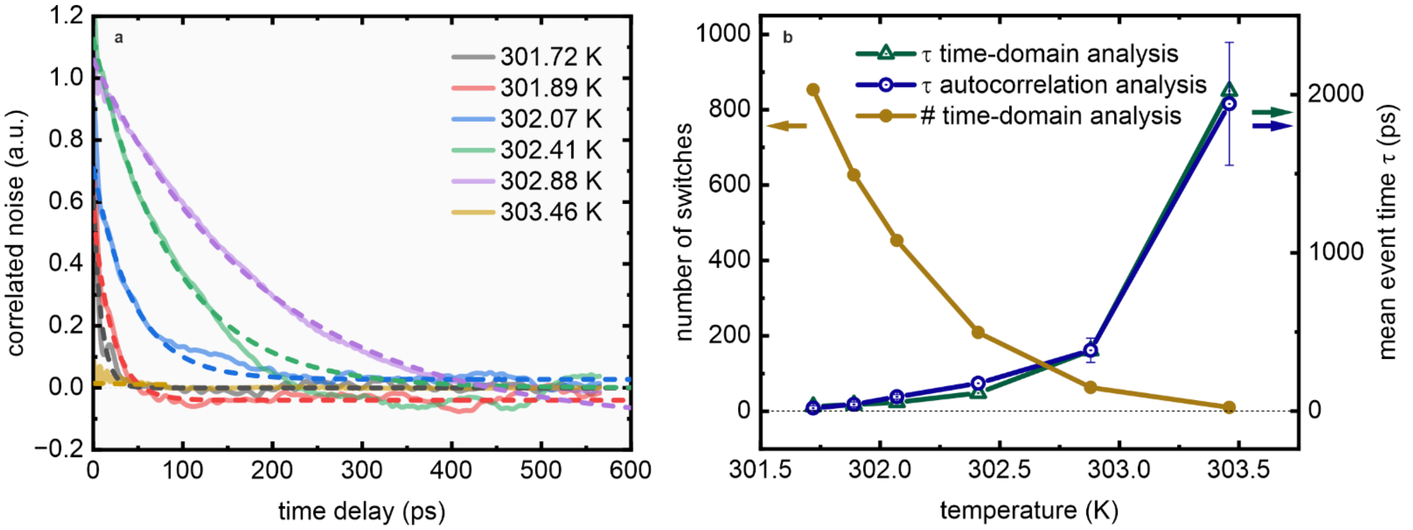

Suppl. Figure 7 | Evaluation of mean dwell time τ of simulated random telegraph noise. a, Exponential fitting of the simulated autocorrelation waveforms to obtain τ as described in the text. **b**, Temperature evolution of the number of switching events (#) in the simulated magnetisation time traces, as well as mean dwell time τ obtained from time-domain analysis and exponential fitting of the autocorrelation function. The error bars are estimated as 20% of the absolute value but at least 15 ps.

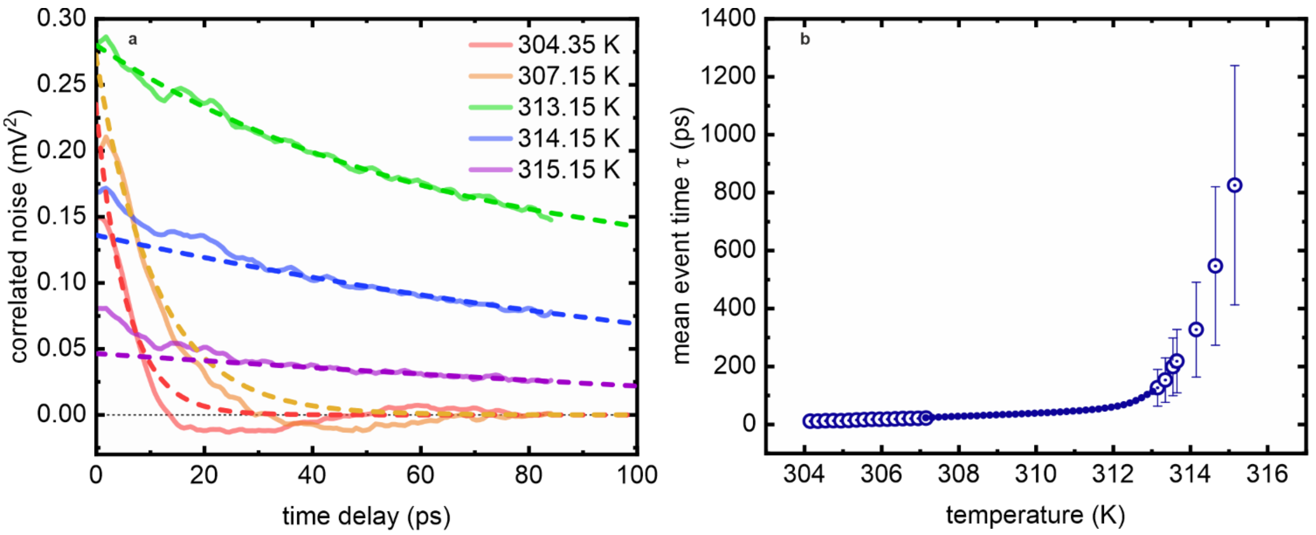

Suppl. Figure 8 | Evaluation of mean dwell time τ of the experimental correlation waveforms. a, Exponential fitting of the experimental autocorrelation waveforms to obtain τ as described in the text. **b**, Temperature evolution of the mean dwell time τ. The error bars are estimated as 30% of the absolute value, but at least 20 ps. The dotted line is a guide to the eye and bridges the temperatures where no meaningful analysis could be conducted due to the background artifact (see Methods).

furthermore neglect the traces where only a marginal number of switches occur within a single simulation run, because here appending of the data sets produces strong artifacts. We find a mean switching time as low as $\tau_{\mathrm{sim}} = 29.50$ ps at 301.72 K, which progressively gets larger as the temperature increases.

Supplementary Figure 7b shows the mean dwell time τ_{sim} obtained by fitting the simulated spin noise correlation waveforms at positive time delays Δt with an exponential decay $\left(\frac{\Delta m_c}{2}\right)^2 e^{-2\frac{\Delta t}{\tau}}$ (Suppl. Fig 7a). We find that the method of extracting τ_{sim} directly from the correlation waveforms shows good agreement to the time-domain analysis of the RTN time traces thus indicating the feasibilty of this approach.

We note that such thermally activated switching processes in bistable systems where two states are separated by an energy barrier ΔE can be described by the Néel-Arrhenius law, which in the context of magnetic systems follows the equation $\tau = \tau_0 e^{\frac{\Delta E}{k_B T}}$ [4]. Here, τ_0 is the attempt time, which is closely related to the intrinsic dynamics of the material system[5,6]. Generally, this formula is used to measure the barrier height ΔE from the temperature dependence of the dwelling time at low temperatures where ΔE is assumed to be temperature independent. Because of the macroscopic energy barriers and exponential temperature dependence, dwelling times are usually very large. In our case, however, both $\Delta \mathrm{E}$ and τ_0 are strongly temperature dependend and ΔE approaches zero at the critical temperature $T_{\mathrm{L,sim}}$. Consequently, the mean dwell time τ_{sim} approaches the frequency of the qF mode, which at this temperature is still in the range of 50 GHz (see Suppl. Fig. 4).

To estimate the mean dwell time τ of the experimental waveforms, we perform the same fitting for multiple temperatures across the SRT. The results are summarised in Suppl. Fig 8. As in the simulated spin noise, τ strongly increases with temperature, suggesting the enhancement of the energy barrier between the two metastable states as the SRT commences. Furthermore, we find dwell times as low as $\tau = 11.21$ ps for the lowest evaluated temperature of 304.35 K indicating that indeed our investigated system shows picosecond random switching. It is interesting to note that the signature of the RTN persists down to temperatures slightly lower than the estimated critical threshold temperature $T_{\mathrm{L}} \sim 305$ K. We assume that this is due to the slight variation of T_{L} by the local magnetic environment within the probe spot due to, e.g., static magnetic domains.

References

1. Yamaguchi, K., Kurihara, T., Minami, Y., Nakajima, M. & Suemoto, T. Terahertz time-domain observation of spin reorientation in orthoferrite $ErFeO_3$ through magnetic free induction decay. *Phys. Rev. Lett.* **110**, 137204 (2013).
2. Balakrishnan, V. *Mathematical Physics: Applications and Problems* (Springer Nature, 2020).
3. Yuzhelevski, Y., Yuzhelevski, M. & Jung, G. Random telegraph noise analysis in time domain. *The Rev. Sci. Instrum.* **71**, 1681–1688, 1150519 (2000).
4. Brown, W. F. Thermal Fluctuations of a Single-Domain Particle. *Phys. Rev.* **130**, 1677–1686 (1963).
5. Hayakawa, K. *et al.* Nanosecond Random Telegraph Noise in In-Plane Magnetic Tunnel Junctions. *Phys. Rev. Lett.* **126**, 117202 (2021).
6. Kanai, S., Hayakawa, K., Ohno, H. & Fukami, S. Theory of relaxation time of stochastic nanomagnets. *Phys. Rev. B* **103**, 094423 (2021).
