# Supplementary material for: Discovery of ultrafast spontaneous spin switching in an antiferromagnet by femtosecond noise correlation spectroscopy
Source: Nat Commun. 2023 Nov 29;14:7651. doi: 10.1038/s41467-023-43318-8 (PMC10687256; doi:10.1038/s41467-023-43318-8)
Supplement: Supplementary file 3 — Description of Additional Supplementary Files [file 41467_2023_43318_MOESM3_ESM.pdf]

Title: Supplementary video 1

Description: High temperature phase, qF mode

Title: Supplementary video 2

Description: High temperature phase, qAF mode

Title: Supplementary video 3

Description: High temperature phase, exchange mode (qAF mode-like)

Title: Supplementary video 4
Description: High temperature phase, exchange mode (qF mode-like)

Title: Supplementary video 5
Description: Low temperature phase, qF mode

Title: Supplementary video 6
Description: Low temperature phase, qAF mode

Title: Supplementary video 7
Description: Low temperature phase, exchange mode (qF mode-like)

Title: Supplementary video 8
Description: Low temperature phase, exchange mode (qAF mode-like)

Supplementary video 9:
Description: Simulated trajectory of magnetization vector at various temperature around spin reorientation transition.
